# Dental Education

**Published:** 1860-07

**Authors:** 


### EDITORIAL DEPARTMENT.

*Dental Education.*—Much discussion has been excited by the various schemes of dental education which have been submitted for the consideration of the profession. Not only this country but England has been divided in sentiment upon this subject, and varying opinions have given rise to diversity of practice. It seems, however, that two dominant ideas of the objects of this education lie at the basis of this multiform disagreement.

There are those who believe that the sole education necessary is a practical training, and they are loud in their commendations of the *practical* as opposed to the *theoretical*. Now, none but a fool will underrate the importance of practical training; the only question is, what shall be considered practical training?

The crude notion of it is that sort of exercise of the hands and eyes which gives steadiness to the one and discernment to the other. It is a mechanical facility which is arrived at, a mere handicraft accuracy which is assured. If the hand be expert to do all it has been taught to do, if the eye is able to recognize all that it has been in the habit of seeing, the training is considered complete. A man reduced to the condition of an automaton which perfectly performs its assigned work upon the application of the foreordained stimulus, is the highest development of this practical training. To our minds, this is not the only desirable achievement of education.

A more lamentably one-sided and imperfect being than the strictly *practical* man is not conceivable by the human mind. He is a man who has traveled perpetually one beaten track, till like the blind mill horse, it is impossible for him to miss it. He would go through all the motions of a given operation with his eyes shut and his mind closed also. His ideas, when he has any, are a mere collection of formulæ. They run in grooves, channeled in his brain, and cannot get out of their accustomed track. Mentally, as well as physically, he has become a machine. The result is, that so long as the old combinations recur in the familiar order, he is a model of manual excellence. The number of times the same motions have been performed, have given a precision to the muscles worthy of commendation, but have left the mind a blank. When new combinations present themselves, the mere *practical* man is completely adrift, without compass or rudder. Never having been in the habit of reasoning upon the phenomena he has witnessed, he is unable to meet any new arrangement of the old appearances, still less to deal with new facts. He stands aghast and bewildered at the unexpected state of affairs, or tries to apply one of his old formulæ, which is utterly inapplicable.

The other idea of education is that which inculcates the necessity of providing the student with principles thoroughly expounded and satisfactorily demonstrated. He is thus armed in advance to meet difficulties. He applies the principles he has acquired to individual cases as they arise. Accustomed to analyse complex phenomena and reduce them to their simplest elements, he is not bewildered by new combinations. The old elements are all present, but under a new form. He appreciates the new arrangement, because familiar with the old, not as a simple but as a compound being; or if a new element is present, he immediately recognizes it because he is already familiar with the old.

The question at issue between the two parties is simply this: Shall dentistry be a manual *art* or shall it be a profession, a branch of the great profession of medicine? If the former, let the practical training be adopted, not, however without the inculcation of principles, for no man who thinks can do anything without a theory. If the latter view be adopted, then let the dentist be so grounded in the fundamental principles of medicine, that he may be able to take his position among medical specialists, and let him be so thoroughly versed in the science of his own specialty, that medical men generally will be compelled to refer to him for what information they may need, not only upon the art but also upon the science of his department.

